# Enhanced photoelectrochemical performance of quantum dot-sensitized solar cell using Cu^2+^ co-doped CdS and CdSe nanoscrystals

**DOI:** 10.1098/rsos.231343

**Published:** 2024-01-31

**Authors:** Thi Tran Anh Tuan, Ha Thanh Tung, Truong Thi Ngoc Chinh, Phan Thanh Hung, Huu Phuc Dang

**Affiliations:** ^1^ School of Basic Sciences, Tra Vinh University, Tra Vinh Province, Vietnam; ^2^ Faculty of Basic Sciences, Vinh Long University of Technology Education, Vinh Long City, Vietnam; ^3^ Faculty of Fundamental Science, Industrial University of Ho Chi Minh City, No. 12 Nguyen Van Bao, Ward 4, Go Vap District, Ho Chi Minh City, 700000, Vietnam

**Keywords:** nanocrystals, quantum dot-sensitized solar cell, high efficiency

## Abstract

Today, nanoscrystals are researched and developed very quickly because of their advantages in many areas of life. One of the potential applications is quantum dot-sensitized solar cells. This is a green, clean, environmentally friendly cell, and has been studied by scientists since 2000. In this study, we fabricated photoanodes with Cu^2+^ ions co-doped into cadmium sulfide (CdS) and cadmium selenide (CdSe) nanoscrystals by successive ionic layer adsorption and reaction, and chemical bath deposition methods to improve absorption spectral intensity of films. The results showed that the absorption intensity increased by eight times compared with our previous results on Cu^2+^ ions doped with CdSe nanoscrystals. The CdS:Cu^2+^ film is optimized at 2% doping, the efficiency is 4.6819%, and the current density is 27.3501 mA.cm^−2^, which is higher when compared with the Cu^2+^ ion only doped into the CdSe quantum dot (19.915 mA.cm^−2^). In addition, the composition of the photoanode was determined by surface and cross-sectional field-emission scanning electron microscope images, and the structure of the film was determined by X-ray diffraction, energy-dispersive X-ray spectroscopy mapping and X-ray photoelectron spectroscopy. Finally, the film's optical properties were studied by ultraviolet-visible spectroscopy, photoluminescence spectroscopy and electrochemical properties by electrochemical impedance spectroscopy. The results obtained have been discussed and presented in great detail.

## 1. Introduction

Dye sensitized solar cells (DSSCs) have been researched and developed by scientists over the past decades, and the efficiency achieved so far is higher than 15% [1,2]. However, this cell has a Shockley–Queisser limitation, so the performance cannot be further increased. This limitation is owing to energy losses by thermal radiation. In addition, another reason is the limitation of the absorption region of dye molecules, so photoanodes cannot absorb all photons in different regions. Since 2000, quantum dot-sensitized solar cells (QDSSCs) have developed extremely strongly, they are studied by many scientists around the world because they are predicted to achieve high efficiency and overcome the Shockley–Queisser limit [3,4]. The nanocrystals are used to replace dye molecules, which can increase the absorption capacity of photoanodes owing to their unique advantages, such as their high absorption coefficient, which can change the absorption region with changing particle size. Inside nanocrystals the multiple exciton generation effect occurs [5,6], that is, an absorbed photon will produce many pairs of excitons. Nanocrystals can also absorb all kinds of photons at different wavelengths.

From the above advantages, nanocrystals have been introduced into photoanodes such as cadmium sulfide (CdS), cadmium selenide (CdSe), lead sufide (PbS), lead selenide (PbSe), cadmium telluride (CdTe), etc. However, the efficiency achieved is not high owing to the limited absorption spectrum of single quantum dot [7–11]. Therefore, scientists are looking for ways to enhance conversion efficiency through the process of combining nanocrystals to form multilayer films using successive ionic layer adsorption and reaction (SILAR) and chemical bath deposition (CBD) methods. The obtained results showed a significant increase in current density and thereby increased efficiency performance [12–14]. At present, there is a method that can increase the current density of the QDSSCs, which is the doping of metals into nanocrystals for multilayer photoanodes such as: Hg doped into PbS [15]; Mn doped into CdS and CdSe achieves 2.5% and 3.77%, efficiency [16,17]; Cu doped into CdSe achieves an efficiency of 4.22% [18]; and Ag doped into CdSe archives an efficiency of 2.72% [19]. The above results are very impressive, with a significant increase in current density. The increase in current density is owing to the appearance of impurity energy levels inside the band gap (*E_g_*) of nanocrystals. However, the above publications have not addressed the problem that metals can be co-doped with nanocrystals such as CdS and CdSe at the same time. Thus, the absorption spectrum of the photoanode can be radically extended, thereby greatly increasing the current density.

In this study, we co-doped Cu metal into CdS and CdSe nanocrystals by SILAR and CBD methods. This study follows on from the group's previous work [18]. We investigated the influence of Cu doping on conversion efficiency, and we optimized the doping concentration at 0.3. The research results on morphology, structure, optical properties and electrochemical properties have been presented and discussed in great detail.

## 2. Experimental section

### 2.1. Materials

Cadmium acetate dihydrate (Cd(CH)_3_COO)_2_.2H_2_O); sodium sulfide non-ahydrate (Na_2_S·9H_2_O); copper (II) sulfate pentahydrate (CuSO_4_.5H_2_O); selenium (Se powder); sodium hydroxide (NaOH); zinc nitrate hexahydrate (Zn(NO_3_)_2_.6H_2_O); sulfur (S powder); potassium chloride (KCl); cuprum (II) chloride dihydrate (CuCl_2_.2H_2_O); zinc nitrate hexahydrate (Zn(NO_3_)_2_.6H_2_O); sodium thiosulfate pentahydrate (Na_2_S_2_O_3_.5H_2_O); acid clorhidric (HCl); ethanol (C_2_H_5_OH); methanol (CH_3_OH); TiO_2_ paste transparent 18NR-T; titanium tetrachloride. Fluorine doped tin oxide (FTO) glass (1.2 × 2.0 cm) was treated with two steps of ultrasonic treatment in a mixture of 0.1 M HCL and ethanol solvent for 30 min to remove dirt, after washing with ethanol and drying. All were bought from Sigma with purity of 99% for analysis.

### 2.2. Fabrication of FTO/TiO_2_/CdS:Cu(x)/CdSe:Cu(0.3)/ZnS photoanode and FTO/Cu_2_S cathode

The FTO/TiO_2_ was synthesized by the TiO_2_ paste screen-printing method (Dyesol, particle size 20 nm, anatase phase), and then the film was heated at 500°C for 30 min. The FTO@TiO_2_@CdS:Cu(x) photoanode (x was the molar ratio between Cu^2+^/Cd^2+^ = 0, 0.1, 0.2, 0.3, 0.5) was synthesized using SILAR method. The FTO@TiO_2_ was immersed in 0.1 M solutions containing Cd^2+^ ions (Cd (CH)_3_COO)_2_.2H_2_O dissolved in 100 ml of ethanol) and Cu^2+^ ions (CuCl_2_ dissolved in 50 ml of the mixture ethanol/distilled water ratio 1 : 1) for 5 min, then cleaned and washed with ethanol. Next, this electrode was immersed in a 0.1 M solution containing S^2−^ ions (Na_2_S.9H_2_O dissolved in 100 ml of distilled water) for 5 min at 60°C, then cleaned and washed with methanol, and dried at 120°C for 15 min. To synthesize an FTO@TiO_2_@CdS:Cu(x)@CdSe:Cu(0.3): the FTO@TiO_2_@CdS:Cu(x) was immersed in 0.1 M solutions (mentioned above) containing ions Cd^2+^ and Cu^2+^ (molar ratio Cu^2+^/Cd^2+^ = 0.3) for 5 min, then cleaned and washed with ethanol. Next, this electrode was dipped in a 0.3 M solution Se^2^- ions (Se powder and Na_2_SO_3_ dissolved in NaOH) for 5 min at 60°C, then removed and washed with distilled water. The number of SILAR cycles was three. The passive layer of ZnS was deposited by two cycles of SILAR coating. The above structure was dipped in a 0.1 M solution of Zn^2+^ ions (Zn (NO_3_)_2_ dissolved in 100 ml of distilled water) for 5 min and cleaned with distilled water. Next, the membrane was immersed in a solution containing 0.1 M S^2−^ ions for 5 min and cleaned with distilled water. The QDSSCs are fabricated by assembling the prepared photoanode with an FTO@Cu_2_S film. Each cell was prepared with an active area of 0.25 cm^2^ and filled with polysulfide electrolyte (0.5 M Na_2_S, 0.2 M S, and 0.2 M KCl in distilled water) and then examined under light approximately 100 mWcm^−2^.

### 2.3. Characterization

The properties of photoanodes and QDSSCs are supported by the following devices. X-ray diffraction spectroscopy (XRD; Philips, PANalytical X'Pert, CuK*α* radiation) used to determine the structure of the material. Ultraviolet-visible (UV-Vis) absorption spectroscopy (JASCO V-670) is used to study the absorbance and transmittance of the film. The electroluminescence spectrophotometer with the function of measuring the total photoluminescence (PL) of different materials, Interface 5000E Electrochemical resistance, it allows the study of the kinetic mechanisms in QDSSCs. The Keithley 2450 photovoltaic system is used to determine the conversion efficiency of the QDSSCs. X-ray photoelectron spectroscopy (XPS) is used to determine the formation of film components based on the binding energy between them. Energy-dispersive X-ray spectroscopy (EDX) and EDX mapping are used to identify the elements present in the film, and scanning electron microscopy (SEM) of the surface and cross section is used to study the photonode with high resolution.

## 3. Results and discussion

**Figure 1.**
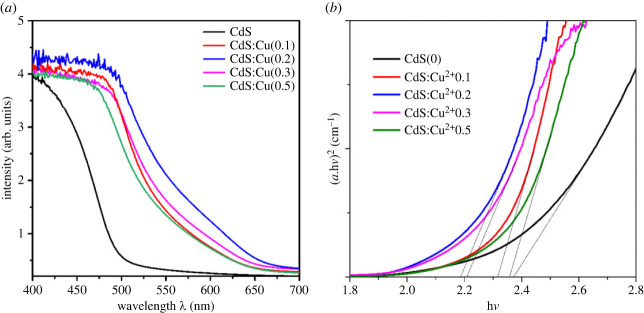
(*a*) Absorption spectrum of Cu^2+^ doped CdS according to different Cu^2+^ ions in FTO@TiO_2_@CdS:Cu^2+^@CdSe:Cu^2+^@ZnS photoanode. (*b*) Graph depicting the relationship between (*α*h*ν*)^2^ and photon energy (h*ν*) from UV–Vis absorption spectrum.

Photoanodes with multilayer structure TiO_2_@CdS: Cu^2+^@CdSe:Cu^2+^@ZnS are fabricated by SILAR and CBD, where CdS:Cu^2+^, CdSe:Cu^2+^ and ZnS nanocrystals are deposited onto the FTO@TiO_2_ semiconductor, and TiO_2_ semiconductors are coated on FTO substrates using screen-printing. The optical properties of the film were recorded by UV–Vis spectroscopy to investigate the influence of Cu^2+^ doping on the counter electrode. Figure 1*a* is the UV–Vis spectrum of a TiO_2_@CdS:Cu^2+^@CdSe:Cu^2+^@ZnS photoanode whose optical absorption varies with the Cu^2+^ ion. The results show that the light absorption strongly depends on the doping concentration of Cu^2+^ ions. Specifically, Cu^2+^ doping in CdS nanocrystals significantly increases the adsorption intensity in the range of 400–700 nm, which is caused by the formation of impurity energy levels inside *E_g_* of the CdS. As we know, pure CdS nanocrystals have an absorption region from ultraviolet to 400 nm, but when doped with Cu^2+^ ions, they are able to absorb photons with wavelengths greater than 400 nm [18]. In addition, the spectral region from 400 nm to 700 nm is the absorption region of CdSe and CdSe:Cu^2+^ nanocrystals, so when doping Cu^2+^ into CdS, there will be both CdS:Cu^2+^ and CdSe:Cu^2+^ materials co-absorbed in the spectral region from 400 nm to up to 700 nm, whereas this is not possible for FTO@TiO_2_@CdS@CdSe:Cu^2+^@ZnS photoanodes. Besides, the absorption edge shift towards the blue light region is owing to the relationship between the quantum confinement effect and the particle size. Numerous factors, including: (i) a high particle size value in comparison to the CdS Bohr radius, (ii) the aggregation of nanomaterials, and (iii) coulomb interactions between charge carriers, can be implicated in the relationship between grain size and *E_g_* value. As a result, the battery's performance can be enhanced as the quantum dot structure's absorption region moves to the visible light region and the concentration of photogenerated electrons rises.

In addition, the formula below calculates the absorption of various photoanode structures:

3.1 $${\left(\alpha hv\right)}^{2}=A.(hv-E_{g})$$

where *α* is the absorption coefficient, *A* is a constant, *ν* is the frequency of the incident radiation, and h is the Planck constant.

Figure 1*b* shows that (*α*h*ν*)^2^ is plotted along the energy axis to determine the *E_g_* of Cu^2+^-doped CdS according to formula (3.1). The estimated values of CdS, CdS:Cu^2+^(0.1), CdS:Cu^2+^(0.2), CdS:Cu^2+^(0.3), CdS:Cu^2+^(0.5) are 2.37 eV, 2.31 eV, 2.18 eV, 2.21 eV and 2.32 eV, respectively. All are lower than *E_g_* (2.4 eV) of pure CdS.

**Figure 2.**
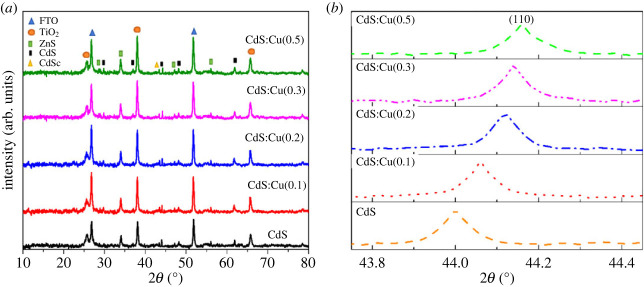
(*a*) XRD spectrum of FTO@TiO_2_@CdS:Cu^2+^@CdSe:Cu(0.3)@ZnS anode and (*b*) the diffraction peak of CdS changes with different doped Cu concentrations.

The XRD spectrum (figure 2*a*) of the FTO@TiO_2_@CdS:Cu@CdSe:Cu(0.3)@ZnS photoanode is under different doped Cu^2+^ concentrations. The diffraction peaks of the SnO_2_ rutile phase and the TiO_2_ anatase phase are also observed in the diffraction spectrum. Characteristics for the anatase phase of TiO_2_ crystals (JCPDS 21-1272) are observed at positions 25.7°; 48.3°; 51.8°; 38.1°, which corresponds to the (101), (200), (211) and (004) planes [20]. Besides, the diffraction peaks of CdS crystals are also found at positions of diffraction angles of 29.80°; 43.9° and 61.80°, respectively, for the (101), (110) and (203) planes, and with the cubic structure (JCPDS 41-1019) [20]. Besides, when increasing the doping concentration of Cu^2+^ affects the position of the diffraction angle 43.9° corresponding to the (110) plane, which is typical for the CdS crystal to shift to a large angle, it proves that there is a substitution of Cd^2+^ by Cu^2+^. In general, alternative doping happens simply when the radius of the doped atoms is lower than the radius of the host atoms. The crystal structure will consequently contract, resulting in a reduction in the separation between the lattice faces. The diffraction peak will move in the direction of the big angle in accordance with Bragg's equation. The Cd atomic radius is 0.097 nm larger than the 0.073 nm Cu atomic radius used in this study. Additionally, the displacement towards a small or large angle might be brought on by the doped atoms' altered effect on the lattice constant, which might indicate the presence of macroscopic residual stress brought on by lattice strain. When compressive stress is applied, some diffraction peaks move towards a large angle and macroscopically residual tension might result in anisotropic lattice contraction. When tensile stress is applied, however, these peaks change to tiny angles. According to standard tag code JCPDS no. 88–2346, two diffraction peaks at diffraction angle position 43.4° correspond to (220) lattice plane of cubic CdSe crystals (zinc blend) [21].

**Figure 3.**
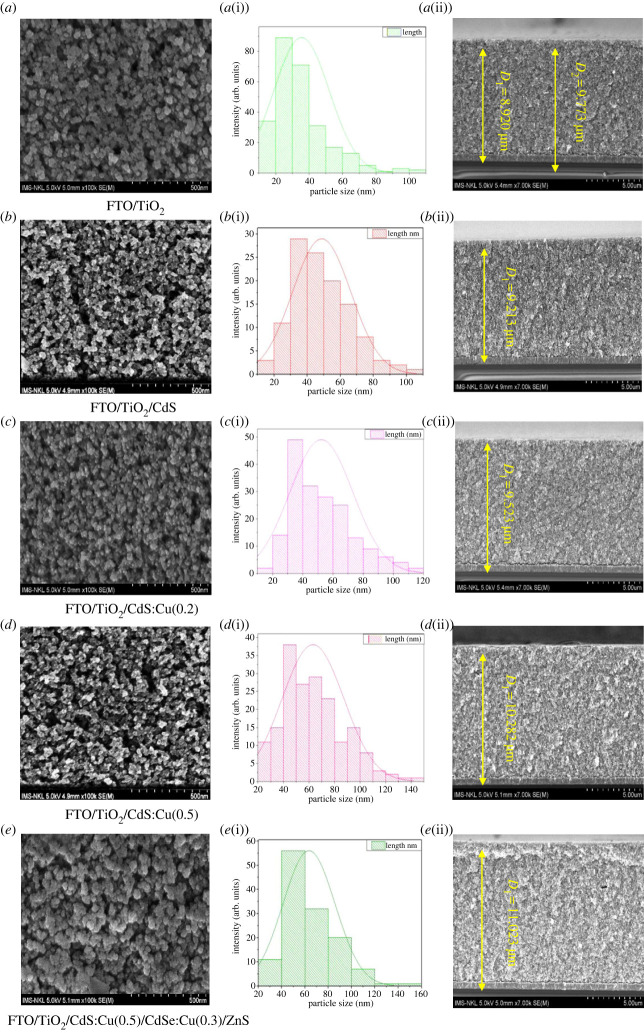
FESEM of (*a*) FTO@TiO_2,_ (*b*) TiO_2_@CdS(0) (*c*) TiO_2_@CdS:Cu(0.5) (*d*) TiO_2_@CdS:Cu(0.2) (*e*) TiO_2_@CdS:Cu(0.2)@CdSe:Cu(0.3)@ZnS.

Figure 3*a* shows the field-emission SEM (FESEM) images of the formed TiO_2_ layer after thermal annealing at 500°C for 30 min. The corresponding size distribution is shown in figure 3*a*(i). The surface morphology of TiO_2_ has no cracks and is uniform with the porous surface. Also, the TiO_2_ particles have a circular shape with a diameter in the range of 20–30 nm. Figure 3*b* observes the CdS layer deposited by SILAR methods, showing that the CdS nanoscrystals attached to the TiO_2_ layer have the form of particles that stick together and have many holes but do not change the surface of TiO_2_. The particle size of the undoped CdS layer is distributed in the range of 40–60 nm. When the Cu^2+^-doped CdS film is at a ratio of 0.2, the particles have a larger size distribution than the undoped film. However, when CdS is doped with Cu^2+^ with a doping concentration of 0.5 (figure 3*d*), the particles tend to clump together with larger sizes and fewer voids compared to 0.2-doped and undoped CdS. Figure 3*e* shows the deposition of CdS, CdSe nanoscrystals coated by a ZnS protective layer; the particle size is very large, 60–80 nm. Also, the thickness of the TiO_2_@CdS:Cu(0.2)@CdSe:Cu(0.3)@ZnS is 11 023 µm.

**Figure 4.**
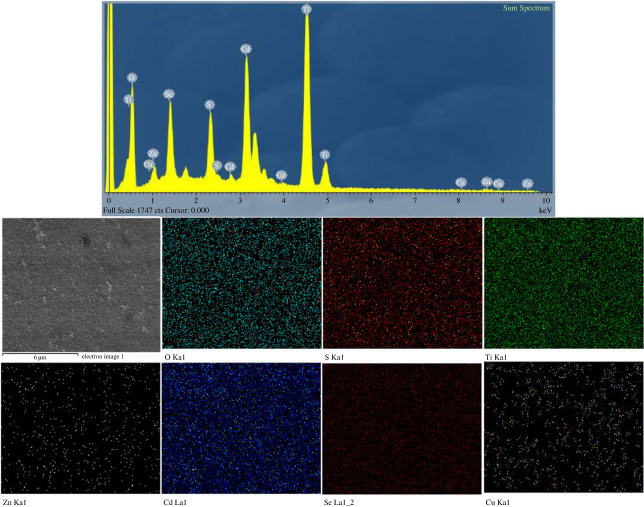
The elemental mapping images of Ti, Cd, Zn, O, S, Se, Cu of the TiO_2_@CdS:Cu(0.2)@CdSe:Cu(0.3)@ZnS photoanode, respectively.

Figure 4 is the EDX spectrum and EDX mapping of elements of the TiO_2_@CdS:Cu(0.2)@CdSe:Cu(0.3)@ZnS photoanode. In general, we can see that all elements are deposited onto the film, including Ti and O from TiO_2_ semiconductors, Cd and S from CdS nanoscrystals, Cd and Se from CdSe nanoscrystals, Cu doped into both CdS and CdSe, and Zn and S from ZnS surface passivation of CdS:Cu^2+^ and CdSe:Cu^2+^ nanoscrystals. This shows that the TiO_2_@CdS:Cu(0.2)@CdSe:Cu(0.3)@ZnS photoanode has been fabricated successfully, which is also supported by surface and cross section XRD and FESEM spectra.

**Figure 5.**
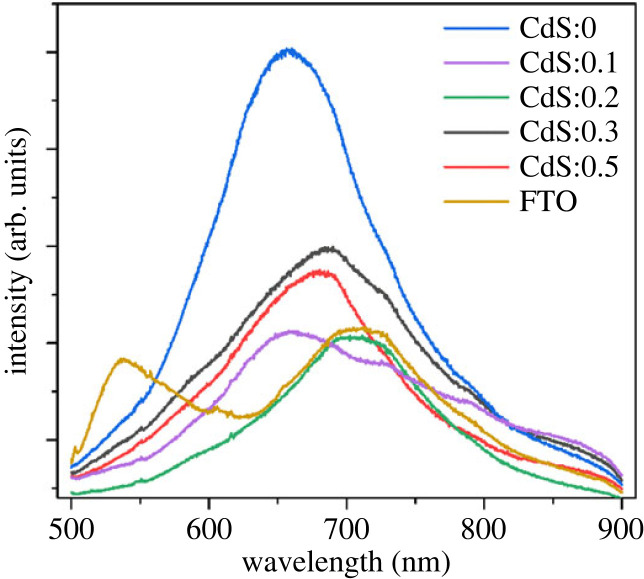
Photoluminescence spectra of FTO@TiO_2_@CdS:Cu@CdSe:Cu(0.3)@ZnS anode structure according to different doped Cu concentrations.

In order to understand the electron injection process from nanocrystals into the TiO_2_ semiconductor, we measured the PL spectrum of the photoanode with Cu concentration varying from 0 to 0.5; this transition requires only 1 picosecond in time [22]. When illuminated, the nanocrystals absorb the photons, which then shift to the conduction band (CB) of nanocrystals and transfer to a TiO_2_ semiconductor when the external circuit potential is greater than that of the exciton pair [23,24]. Figure 5 depicts the PL spectra of TiO_2_@CdS:Cu@CdSe:Cu(0.3)@ZnS photoanodes with doped Cu concentrations at different ratios. In general, all photoanodes with Cu concentrations from 0.1 to 0.5 have less luminescence intensity than TiO_2_@CdS@ZnS films. The reason is that a part of the electrons in the CB of the radiated CdS shift to the Cu energy levels in *E_g_* of the CdS quantum dot, which does not cause luminescence [23]. In general, there is an energy shift between the UV–Vis spectrum peak and the PL spectrum corresponding to CdS(0.2), 175 nm∼11.4 eV. The reason is owing to the Stokes transitions that exist at the state surfaces of QDs. Surface states are found to be the main cause of rapid recombination of excited electrons [25]. When electrons receive energy the size from the valence band to the conduction band and then back immediately recombine with the surface states inside the empty band of QDs. Experiments show that the Stokes transition is a difference between energy levels, the atomic basis is to carry out the conversion between the energies of this process using thermodynamics. The result is a peak shifted PL diffusion phenomenon with a peak absorption spectrum [26]. Furthermore, the lines are also made dark in the cellular configuration of the exciton boundary region [26].

**Figure 6.**
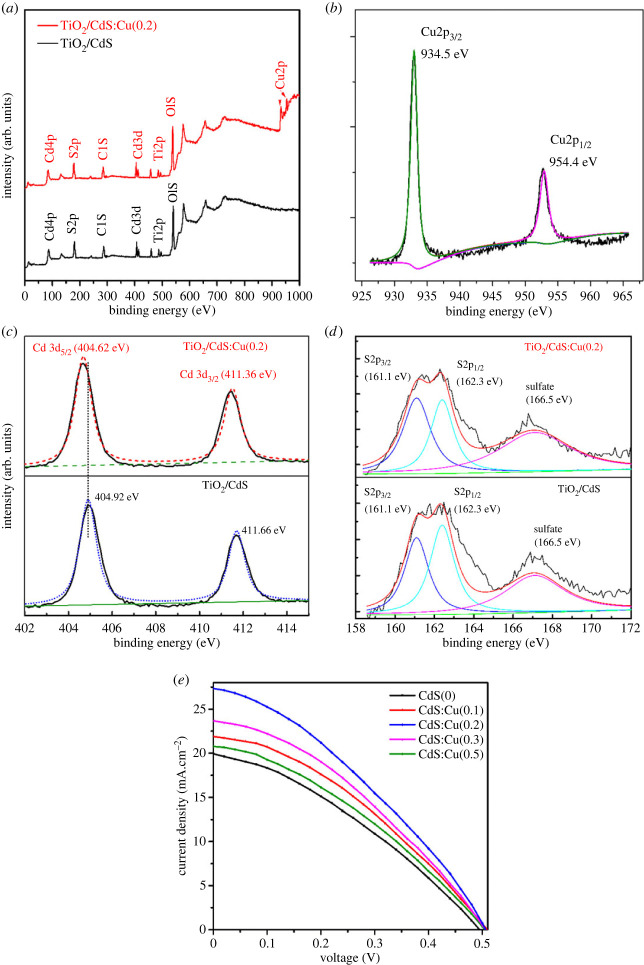
XPS spectra of TiO_2_/CdS and TiO_2_/CdS: Cu (0.2): (*a*) survey spectrum and high-resolution spectra of (*b*) Cu2p, (*c*) Cd3d, (*d*) S2p and (*e*) efficiency performance of Cu^2+^ doped CdS according to different doped Cu^2+^ ions in FTO@TiO_2_@CdS@CdSe:Cu^2+^@ZnS anode.

The valence states of the elements in the photoanodes TiO_2_@CdS and TiO_2_@CdS:Cu(0.2) were analysed using XPS. Figure 6*a* displays the XPS of TiO_2_@CdS and TiO_2_@CdS:Cu(0.2) as obtained from the survey spectrum. The observation revealed the presence of titanium (Ti), cadmium (Cd), sulfur (S), copper (Cu), and oxygen (O) elements. Figure 6*b,c*, and *d* display the high-resolution XPS of copper (Cu), cadmium (Cd) and sulfur (S) elements, respectively. Moreover, it is important to note that all peaks were calibrated using the C1s peak at 284.8 eV. Figure 6*b* displays two distinct peaks at energy levels of 934.5 and 954.4 eV, corresponding to the Cu2p_1/2_ and Cu2p_3/2_ orbitals, respectively. These peaks identify the chemical state of Cu^2+^ ions [27]. Figure 6*c* illustrates the presence of a doublet configuration characterized by the Cd 3d_5/2_ and Cd 3d_3/2_ peaks, which are observed at 404.98 and 411.68 eV, respectively, [28]. This observation suggests the formation of chemical bonds between Cd^2+^ cations and S^2−^ anions in the CdS nanocrystals. Furthermore, it is observed that there is a minimal shift following Cu doping. The deconvolution of the S2p peak, as depicted in figure 6*d*, reveals a spin-orbit doublet of the S2p orbital, which splits into 2p3/2 (161.1 eV) and 2p1/2 (162.3 eV). This observation provides confirmation of the existence of Cd-S bonding [29]. The presence of additional peaks at an energy level of 166.5 eV can be attributed to the presence of sulfate on the surface [18].

Figure 6 shows the efficiency characteristics with different TiO_2_@CdS:Cu^2+^@CdSe:Cu^2+^(0.3)@ZnS photoanodes. The photovoltaic parameters of QDSSCs are also shown in table 1. It is shown that the open-circuit potential values of different but similar structures have approximately the same results. The obtained efficiency varies with different doping concentration; the highest efficiency is 4.6819%, *V*_oc_ is 0.5 V, *J*_sc_ is 27.3501 mA.cm^−2^, fill factor (FF) is 0.3523 for QDSSCs with 0.2 Cu^2+^ ions. The efficiency decreases sharply when the concentration is less than or greater than 0.2. The above results show that although CdS is a buffer layer in the counter electrode, the doping of Cu into CdS has a great influence on the photogenerated current density, thus affecting the efficiency performance. This structure extends the absorption peak of CdS to the 400–500 nm region, this region has the absorption of both materials, CdS:Cu^2+^ and CdSe:Cu^2+^, so that photons in this region are thoroughly absorbed and transfer electrons to the CB of TiO_2_. The CB level of the CdS quantum dot has a lower value of its oxidizing capacity than the CdSe quantum dot; therefore, the presence of induced CdS nanocrystals prevents photo-oxidation and reduces surface defects in CdSe nanocrystals. Also, the *E_g_* and CB position of the ZnS passive layer are 3.6 eV and −3.0 eV, respectively. Therefore, the CB of ZnS is more negative than that of the nanocrystals, thereby reducing the back-propagation of the photogenerated electrons of nanocrystals and TiO_2_ with the oxidants of the electrolyte. The holes are transferred from the nanocrystals to the electrolyte by the ZnS passivation layer. Accordingly, this hole transfer facilitates hole and electron separation and electron transfer into the TiO_2_ CB. In addition, CdS doped with Cu^2+^ increases light absorption, as observed in the UV–Vis spectrum. In this case, a wide excitation wavelength band will provide more photons, and thus a higher current density is achieved. On the other hand, doping Cu^2+^ into the CdS nanocrystals leads to the restriction of excitons recombination, as observed in the PL spectra.

**Table 1.** A comparison of QSSCs fabrication results. (PCE, power conversion efficiency.)

QDSSCs	methods	counter electrodes	PCE (%)	ref
CdS/Mn-CdSe	SILAR, CBD	Cu_2_S	4.9	[30]
CdS_x_Se_1−x_/Mn-CdS	SILAR, CBD	Cu_1.8_/CuS	3.26	[31]
CdS/CdSe:Cu	SILAR	Cu_2_S	4.22	[18]
CdS/Mn-CdSe	SILAR	Cu_2_S	3.8	[32]
CdS:Cu^2+^/CdSe:Cu^2+^/ZnS	SILAR, CBD	Cu_2_S	4.6819	present work

From table 1, we see that the results of manufacturing QDSSCs based on co-doping Cu into CdS and CdSe have brought positive results when compared with other results in table 1. There is a very significant increase in strong performance at 4.6819% when compared with the results of the research group when only doping Cu into CdSe (4.22%) [18].

**Figure 7.**
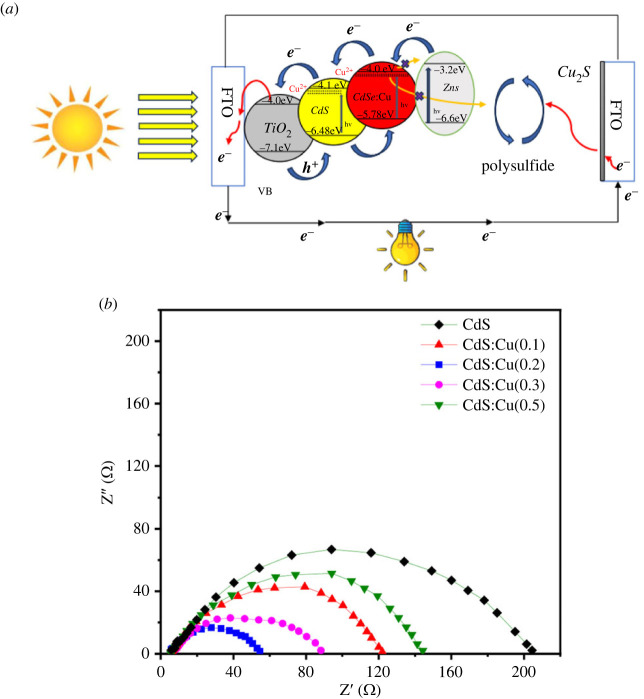
(*a*) A diagram structure and energy level positions of QDSSCs [18,33–35] and (*b*) electrochemical impedance spectroscopy to determine resistance values related to the electron transition processes in the QDSSCs.

The electrochemical impedance spectra obtained from five values of Cu-doped concentration in CdS nanocrystals are used to study the kinetic processes occurring in QDSSCs. Basically, the experimental spectrum after being measured are fitted according to the circuit model of QDSSCs to determine two basic resistance values, which are *R*_ct1_ and *R*_ct2_. *R*_ct1_ is the resistance against electrons as they diffuse in the Cu_2_S cathode and Cu_2_S/electrolyte surface. Meanwhile, *R*_ct2_ is the resistance against the charges transfer flow between the TiO_2_/nanocrystals surface, and the diffusion process in the TiO_2_ film. In general, the smaller the resistance values, the smaller the recombination losses, and the greater the current density, the results of which are presented in table 2 and figure 7. Figure 7*a* is a structure of QDSSCs that describes the electron transitions in the QDSSCs components.

**Table 2.** Parameters characterizing of the QDSSCs. (PCE, power conversion efficiency.)

%Cu	*E_g_* (eV)	*V*_oc_ (V)	*J*_sc_ (mA.cm^−2^)	FF	PCE (%)	*R*_ct1_ (Ω)	*R*_ct2_ (Ω)
*X* = 0	2.38	0.48	19.9305	0.3453	3.3050	12.04	186.4
*X* = 0.1	2.32	0.5	21.8928	0.3813	3.9602	11.08	143.2
*X* = 0.2	2.18	0.5	27.3501	0.3523	4.6819	10.58	24.78
*X* = 0.3	2.21	0.5	23.6811	0.3478	4.4182	10.88	55.56
*X* = 0.5	2.35	0.5	20.7712	0.3471	3.6121	11.32	153.2

From table 1 and figure 7*b*, we see that the *R*_ct2_ and *R*_ct1_ resistances of 0.2 Cu doped in CdS nanocrystals of QDSSCs on the basis of FTO@TiO_2_@CdS:Cu@CdSe:Cu(0.3)@ZnS electrodes are the smallest at 24.78 Ω and 10.58 Ω, respectively, as compared with other doped concentrations of Cu. This filling leads to the reduction of electron assembly processes owing to the rapid transition of electrons across the TiO_2_, nanocrystals and Cu_2_S films and the TiO_2_/nanocrystals, Cu_2_S/electrolyte surfaces. This result is also reflected in the sharp increase in *J*_sc_ of 27.3501 mA.cm^−2^. Cu doping in CdS increases the possibility of light harvesting, generation, collection and injection in QDSSCs, which is also confirmed by the results of the absorption spectrum, which show a much higher enhancement of the absorption spectrum as compared with the results of Phat *et al*. [18]. The resistance values *R*_ct2_ and *R*_ct1_ increase when the Cu doping concentration is greater or less than 0.2. The case of doping concentrations of 0 and 0.1 gives large *R*_ct2_ resistance values of 186.4 Ω and 143.2 *Ω*, respectively. The reason is that the resistance of Cd is larger than that of Cu in the CdS and CdS:Cu^2+^ films. This result is also completely consistent with the results obtained from the absorption spectrum and the FESEM results at a Cu doping concentration greater than 0.2. The doping at high concentration makes the CdS:Cu^2+^ film no longer homogeneous, leading to agglomeration to form larger particles. The results of this study are also consistent with those of Mohammed *et al*. [36]; the authors doped Cu into CdS in QDSSCs with a TiO_2_@CdS:Cu electrode and obtained an extension of the absorption peak and an enhancement of the conversion efficiency.

## 4. Conclusion

In summary, QDSSCs based on FTO@TiO_2_@CdS:Cu@CdSe:Cu(0.3)@ZnS photoanodes with doping concentrations of Cu varying from 0, 0.1, 0.2, 0.3 and 0.5 have been successfully fabricated by SILLAR, CBD and screen-printing methods. The structure of the FTO@TiO_2_@CdS:Cu^2+^@CdSe:Cu^2+^(0.3)@ZnS photoanode has been determined by XRD, XPS and EDX mapping, and the optical properties of the photoanodes are measured by UV–Vis and PL spectroscopy to evaluate the enhancement in intensity and broadening of the absorption peak and at the same time demonstrate the presence of impurity Cu in the CdS host. The results of measuring conversion efficiency increased to 4.6819%, *V*_oc_ to 0.5 V, *J*_sc_ to 27.3501 mA.cm^−2^, FF to 0.3523. This result is much higher than FTO@TiO_2_@CdS@CdSe@ZnS (2.07%) and FTO@TiO_2_@CdS@CdS(0.3)@ZnS photoanodes (4.22%), especially the sharp increase of the current density up to 27.3501 mA.cm^−2^. This is owing to the great absorption of photons present between 400 nm and 550 nm from the CdS:Cu^2+^ and CdSe:Cu^2+^ nanoscrystals.

## Data Availability

Data are available from the Dryad Digital Repository: https://doi.org/10.5061/dryad.gmsbcc2v8 [37].
